# Supplementary material for: Schedule-Dependent Treatment Increases Chemotherapy Efficacy in Malignant Pleural Mesothelioma
Source: Int J Mol Sci. 2022 Oct 8;23(19):11949. doi: 10.3390/ijms231911949 (PMC9569655; doi:10.3390/ijms231911949)

Supplementary Figure S1

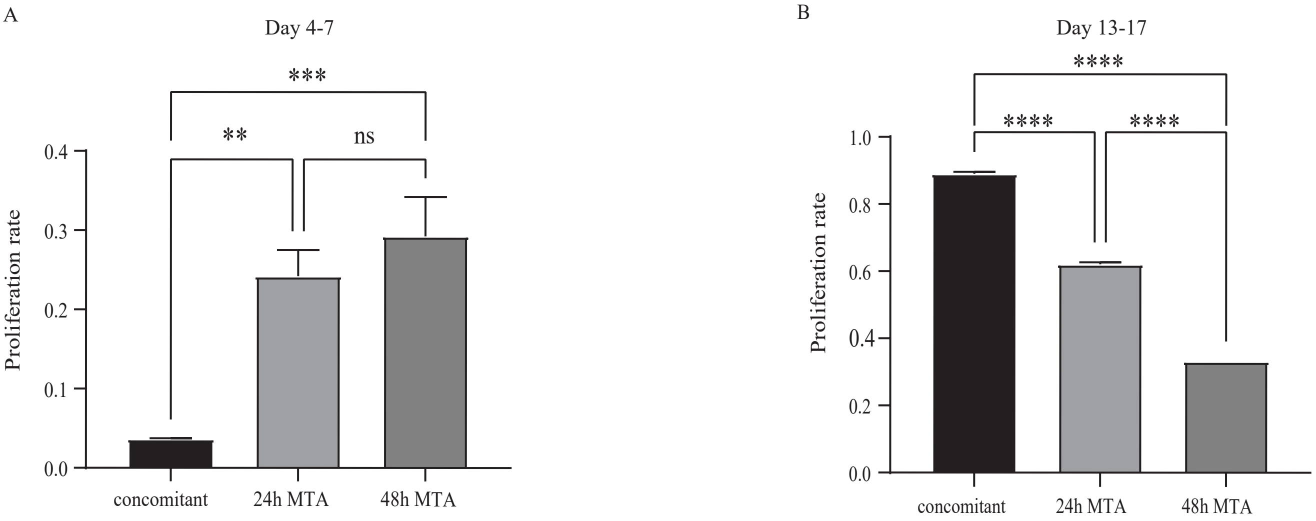

Supplementary Figure S2a

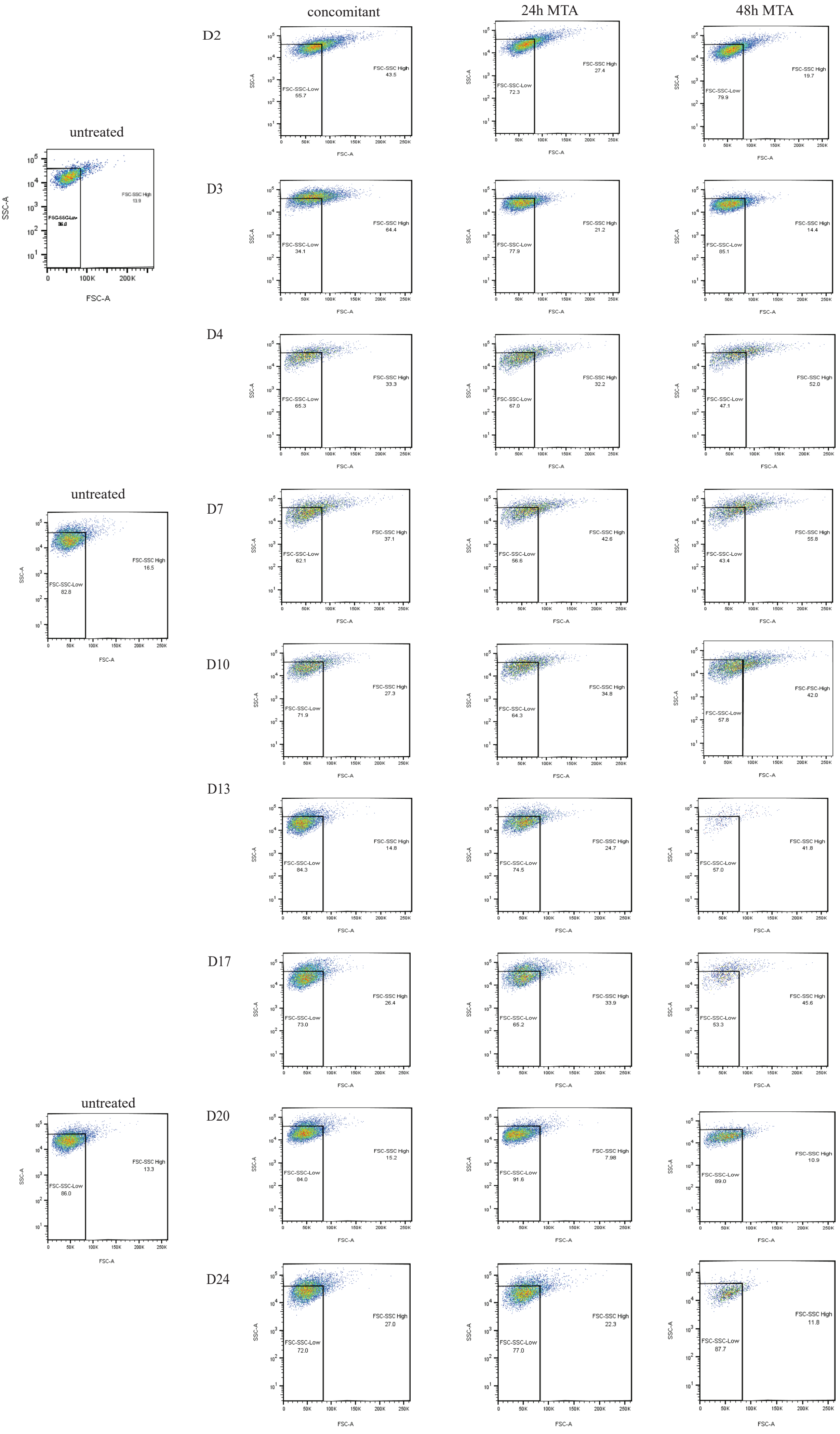

Supplementary Figure S2b

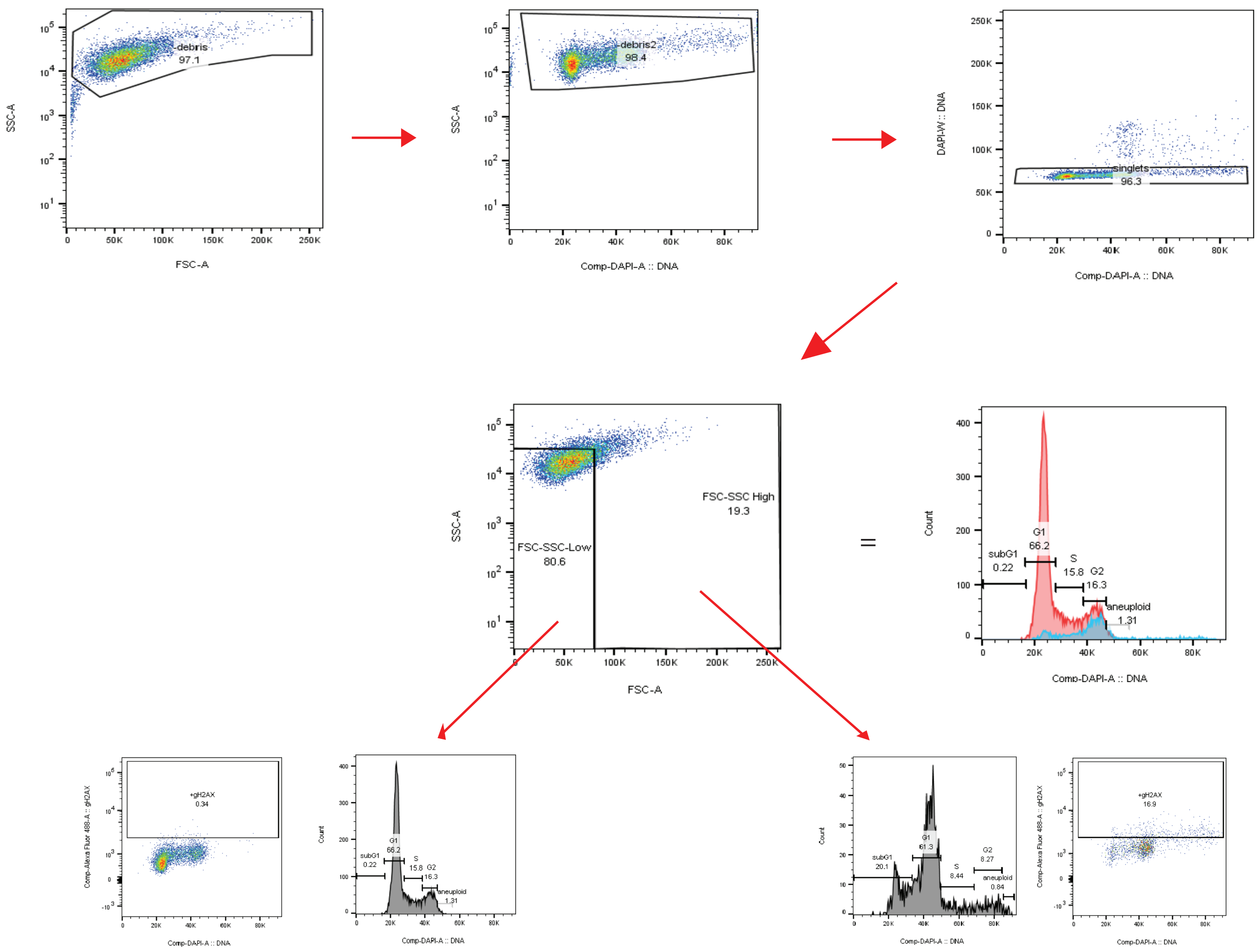

Supplementary Figure S3

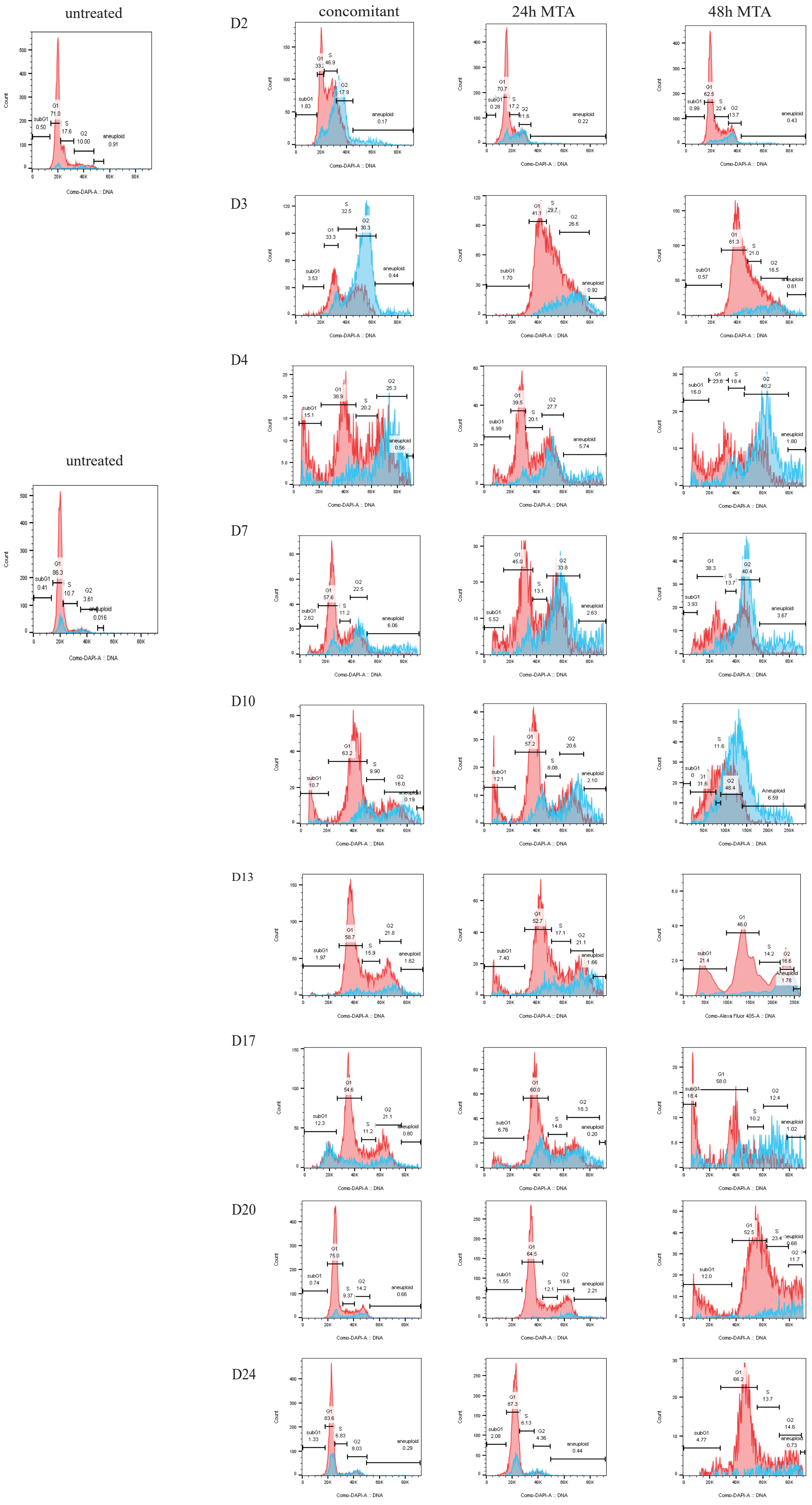

Supplementary Figure S4

A

Untreated

1h 1mM H2O2

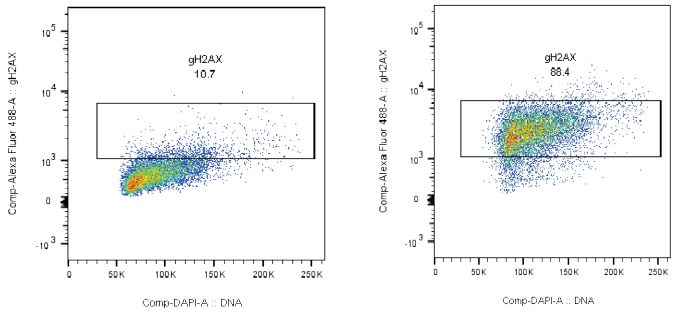

B

Untreated

24h 5µM cisPt

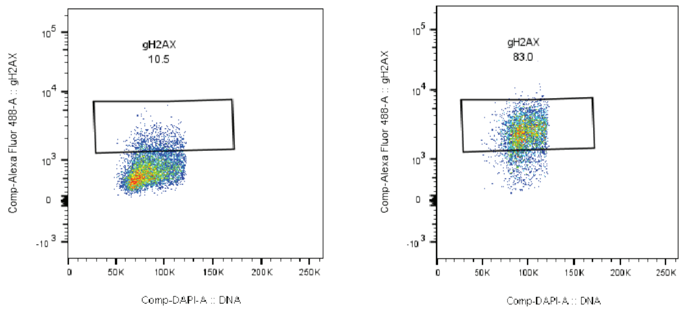

Supplement: Supplementary file 1 [file ijms-23-11949-s001.zip › ijms-1915958-supplementary.pdf]
